# Catalytic Activity of Piezoelectric and Paraelectric BaTiO_3_ Nanoparticles

**DOI:** 10.3390/nano16161008

**Published:** 2026-08-17

**Authors:** Akram Asadi, Hossein Kalhori, Andrea A. Greschner, Andreas Ruediger, Alain Pignolet

**Affiliations:** 1 Centre Énergie Matériaux Télécommunications, INRS—Institut National de la Recherche Scientifique, 1650 Boulevard Lionel-Boulet, Varennes, QC J3X 1P7, Canada; akram.asadi@inrs.ca (A.A.); hossein.kalhori@polymtl.ca (H.K.); andrea.greschner@inrs.ca (A.A.G.); andreas.ruediger@inrs.ca (A.R.); 2 Process Engineering Advanced Research Lab (PEARL), Department of Chemical Engineering, Polytechnique Montreal, Station Centre-Ville, Montreal, QC H3C 3A7, Canada

**Keywords:** barium titanate (BaTiO_3_), piezoelectric materials, spontaneous polarization, ferroelectric–paraelectric transition, nanoparticles, catalytic activity

## Abstract

Ultrasonically assisted catalysis—also called sonocatalysis—is one of the advanced oxidation processes used for wastewater treatment. It has been shown that using nanoparticles of piezoelectric materials as nanocatalysts in sonocatalysis greatly enhances the catalytic reaction rates, a phenomenon that has been dubbed piezocatalysis. Since both sonocatalysis and piezocatalysis are excited by ultrasonic waves and occur simultaneously, it is challenging to discriminate between these two processes and to quantify the contribution of the material’s piezoelectric properties to the overall catalytic activity. It has been previously reported that the piezoelectric properties of the catalyst nanoparticles can improve their catalytic activities by up to one order of magnitude. In this study, we compare the catalytic activity of nanoparticles of both ferroelectric and paraelectric BaTiO_3_, hence piezoelectric and non-piezoelectric BaTiO_3_ nanoparticles. BaTiO_3_ nanoparticles of two different sizes were synthesized using a microwave-assisted hydrothermal method. After a full characterization by transmission electron microscopy (TEM), X-ray diffraction (XRD), and temperature-dependent Raman spectroscopy, the catalytic activities of the BaTiO_3_ nanoparticles were determined by monitoring the time dependence of the optical absorption of a solution containing the model pollutant methyl orange, to which the dispersed piezoelectric BaTiO_3_ particles were added as catalysts. The 50 nm nanoparticles were found to have a tetragonal crystal structure and symmetry and to be piezoelectric, while the 10 nm nanoparticles had a cubic crystal structure and symmetry and exhibited no piezoelectricity. This study reveals that non-piezoelectric BaTiO_3_ nanoparticles exhibit a moderate catalytic activity for the degradation of methyl orange, similar to that of non-piezoelectric TiO_2_ nanoparticles. Furthermore, it also shows that, at room temperature, 90% of the overall catalytic activity of piezoelectric BaTiO_3_ nanoparticles is due to piezocatalysis, while the remaining 10% is related to sonocatalysis. Using liquid chromatography coupled to mass spectrometry (LC-MS), possible chemical decomposition pathways of the methyl orange dye have also been suggested.

## 1. Introduction

Water is a fundamental resource essential for all forms of life. However, rapid industrialization, agricultural expansion, and population growth have significantly contributed to water pollution. Various contaminants, including pesticides, phenols, heavy metals (e.g., As (V), Hg (II), and Cr (VI)), harmful microorganisms, and organic dyes, are commonly found in bodies of water. Among these pollutants, the widespread use of organic dyes in textile, paper, food, and cosmetic industries, as well as their persistence, toxicity, and resistance to conventional wastewater treatment methods, is a known environmental hazard [1,2]. In addition to causing severe environmental damage, these pollutants are also a serious health hazard, affecting vital organs such as the kidneys, lungs, skin, and brain. According to the World Health Organization (WHO), permissible concentrations of organic dyes in drinking water should not exceed 1 ppm [3]. Therefore, addressing water contamination is critical to mitigating its adverse effects and ensuring the availability of clean water resources [4].

In response to growing environmental concerns, significant efforts have been made to develop efficient wastewater treatment technologies. Among the advanced oxidation processes used to treat wastewater, piezocatalysis is an emerging approach based on the piezoelectric properties of the material used as a catalyst. When using the mechanical energy of ultrasonication to drive catalytic reactions, piezocatalysis acts in parallel with sonocatalysis, significantly enhancing the sonocatalytic reaction rates. Piezocatalysis has garnered significant attention for its potential in both environmental remediation and clean energy generation [5,6]. This technique exploits the piezoelectric effect, wherein non-centrosymmetric materials convert mechanical energy into electrical energy. Piezoelectric materials have been suggested, or are being used, to convert various mechanical forces or vibrations originating from friction, wind, and tidal movements into electricity. Due to their non-centrosymmetric crystal structure, piezoelectric materials develop a strain-induced net dipole moment under external mechanical force, as the centers of positive and negative charges in the unit cell shift out of alignment. In contrast to the piezoelectric crystals, paraelectric materials with a centrosymmetric crystal structure do not show any response to external forces, meaning that no polarization is induced by applying stress. Although the piezoelectric effect has been known for over a century, its application in nanomaterial-based catalysis has only recently been explored [7,8]. Indeed, piezoelectricity also presents a promising pathway for sustainable water treatment [9] as the lattice deformation-produced dielectric polarization has been shown to facilitate catalytic reactions [10,11,12].

Many piezoelectric materials have been reported to exhibit a piezocatalysis effect, including lead zirconate titanate (PZT) and lead-free ZnO, BiFeO_3_, and BaTiO_3_, which are growing in popularity [13]. Among these lead-free candidates, barium titanate (BaTiO_3_) exhibits significant spontaneous polarization, excellent piezoelectric properties, and has shown good performance in wastewater treatment [14,15]. At room temperature, BaTiO_3_ is tetragonal and piezoelectric, and at a temperature around 120 °C, it undergoes a phase transition to a non-polar cubic paraelectric phase, which consequently is not piezoelectric [16].

In a previous study, we estimated the contribution of piezoelectricity to catalytic activity by comparing the decomposition of organic dyes using BaTiO_3_ and TiO_2_ nanoparticles as catalysts, exploiting the structural similarity between the surface of anatase TiO_2_ and the active TiO_2_-terminated surface of BaTiO_3_, while accounting for the fact that BaO-terminated surfaces of BaTiO_3_ are catalytically inactive. However, BaTiO_3_ and TiO_2_ are different materials, and catalysis being surface-dependent, the surfaces of TiO_2_-terminated BaTiO_3_ and anatase TiO_2_ are not the same.

The objective of this work was to use a unique catalyst material in its piezoelectric and non-piezoelectric phases to quantify the specific contribution of its piezoelectric properties to the overall catalytic activity. Therefore, we compare the catalytic activity of (i) piezoelectric BaTiO_3_ nanoparticles and (ii) non-piezoelectric BaTiO_3_ nanoparticles for the degradation of methyl orange (MO), an organic dye commonly used in the textile industry and often found in wastewater. Hence, methyl orange was selected as a model pollutant in this study [17].

It is known that the polar properties of ferroelectric materials are size-dependent [18]. In particular, the Curie temperature of BaTiO_3_, the temperature at which there is a transition from the ferroelectric (hence piezoelectric) tetragonal phase to the paraelectric (hence non-piezoelectric) cubic phase, decreases when the particle size decreases [19]. We investigated and compared the catalytic activities of BaTiO_3_ nanoparticles 50 nm in size, which are piezoelectric at room temperature, and BaTiO_3_ nanoparticles 10 nm in size, whose Curie temperature is below room temperature and hence are non-piezoelectric at room temperature. This comparison highlights how the presence or absence of piezoelectric properties in the chosen catalyst impacts their catalytic activity.

Our findings reveal that the ferroelectric phase of the barium titanate nanoparticles has significantly enhanced catalytic efficiency at room temperature compared to the paraelectric phase. Beyond the active surface increase when particle sizes decrease, the size and temperature dependence of the BaTiO_3_ nanocatalysts’ properties, whether they are piezoelectric or not, enable the separation of sonocatalytic and piezocatalytic contributions. Our results also emphasize the beneficial piezocatalytic properties of BaTiO_3_, offering valuable insights into new ways to choose catalysts and to optimize their catalytic properties for wastewater treatment applications [20,21,22].

## 2. Materials and Methods

### 2.1. Materials and Synthesis of BaTiO_3_ Powders

Methyl orange (C_14_H_14_N_3_NaO_3_S) and commercial BaTiO_3_ powder with grains of a nominal size of 50 nm were purchased from Sigma-Aldrich (MilliporeSigma Canada Ltd., Oakville, ON, Canada). The BaTiO_3_-10 nm powders with a narrow size distribution were synthesized using a microwave-assisted hydrothermal method. For this purpose, analytical-grade precursors of titanium butoxide (>97%), barium hydroxide octahydrate (>98%), hydrogen peroxide (30% wt), ethanol (>99%), and potassium hydroxide (≥85%) from Sigma-Aldrich chemicals, and oleic acid (≥90%) from Alfa Aesar (now Thermo Scientific Chemicals, Ottawa, ON, Canada) chemicals were employed. In total, 50 mmol of titanium butoxide was added to 5 mL of ethanol under constant magnetic stirring and served as the source for titanium. After 10 min, 75 mmol of Ba(OH)_2_·8H_2_O dissolved in 5 mL of preheated distilled water (>90 °C) was added to the mixture along with 2 mL of H_2_O_2_ acting as a hydrogen scavenger. To avoid instant coagulation of titanium and prevent further growth, a surfactant was used. Then, 1.5 mL of oleic acid was added dropwise, and the pH of the mixture was adjusted to 13 using a 5 M KOH solution. The homogeneity of the solution was ensured by stirring for an additional 10 min before transferring to a 23 mL Teflon container. This container was then sealed in a microwave acid digestion PTFE autoclave from Parr Instrument (Moline, IL, USA) and placed in a Panasonic Inverter Microwave Oven (2.45 GHz) operated at 360 W for 4 min. The autoclave was cooled down to ambient temperature, and the resulting product was washed in preheated distilled water before drying in an oven at 80 °C for 15 h or more [23,24].

### 2.2. Characterization

The phase identification and crystal structure of the BaTiO_3_ nanoparticles were determined using XRD (Bruker D8 Advance diffractometer; Bruker AXS LLC, Madison, WI, USA) with Cu Kα radiation (λ = 1.5406 Å). The measurements were performed at room temperature, using a 0.02° step size from 20 to 60°. Bright-field images of the nanoparticles were acquired with a transmission electron microscope (TEM, JEOL Model: JEM-2100F; JEOL USA, Inc., Peabody, MA, USA) to assess the nanoparticles’ size and morphology. Furthermore, Raman and temperature-dependent Raman measurements were performed using a Horiba iHR320 system (HORIBA Canada, Inc., Burlington, ON, Canada) equipped with a thermoelectrically cooled Horiba Scientific Synapse Back-Illuminated Deep Depletion CCD detector and a 473 nm (linearly polarized, TEM00) solid-state blue Cobolt 04-01 laser (HÜBNER Photonics GmbH, Kassel, Germany) used as the excitation source. The laser was operated at a low power (0.75 mW) to avoid thermally induced effects on the samples. A Linkam THMS600 stage (Linkam Scientific Instruments, Redhill, UK) was used for all the acquired temperature-dependent Raman measurements.

The samples to be analyzed consisted of sealed vials with water contaminated with 4 mg/L of dissolved methyl orange dye and containing a concentration of 1 g/L of BaTiO_3_ nanoparticles as catalysts. They were completely submerged and underwent ultrasonication in an 80 W ultrasonic bath (Branson CPXH ultrasonic bath system; BRANSON Ultrsonics Corporation, Subsidiary of Emerson Electric, Brookfield, CT, USA) operating at a frequency of 40 kHz for different durations. The temperature was meticulously regulated using a submersible heating device (George Ulanet 335-115 Heat-O-Matic Immersion Heater; Ulanet—a Vernatherm by Vernet Product Line, Columbus, IN, USA). The dye-contaminated samples submitted to different times of ultrasonication were subsequently also analyzed by high-performance liquid chromatography-quadrupole time-of-flight mass spectrometry (HPLC-QqTOF-MS) on a Shimadzu Nexera UHPLC (Shimadzu Scientific Instruments, Columbia, MD, USA) coupled with a Sciex (Concord, ON, Canada) Triple TOF 5600+ system. Separation of components was performed at 40 °C and a flow rate of 0.3 mL min^−1^ on a Phenomenex (Torrance, CA, USA) Biphenyl column (2.1 × 50 mm, 1.7 µm) with mobile phases of water (A) and acetonitrile (B), both containing 0.1% formic acid. The elution gradient used was as follows: 5% B held for 1 min, then increased linearly to 40% B at 15 min, up to 95% B at 15.5 min. TOF-MS and MS/MS analysis were performed using electrospray ionization (ESI) in negative mode with the mass ranges of *m*/*z* 118–970 and *m*/*z* 40–800, respectively. To ensure accurate mass measurements, the instrument was automatically calibrated every four injections with a mix of standard compounds covering the range *m*/*z* 118–966.

## 3. Results and Discussion

### 3.1. Morphology and Structural Characterizations

**Figure 1:**
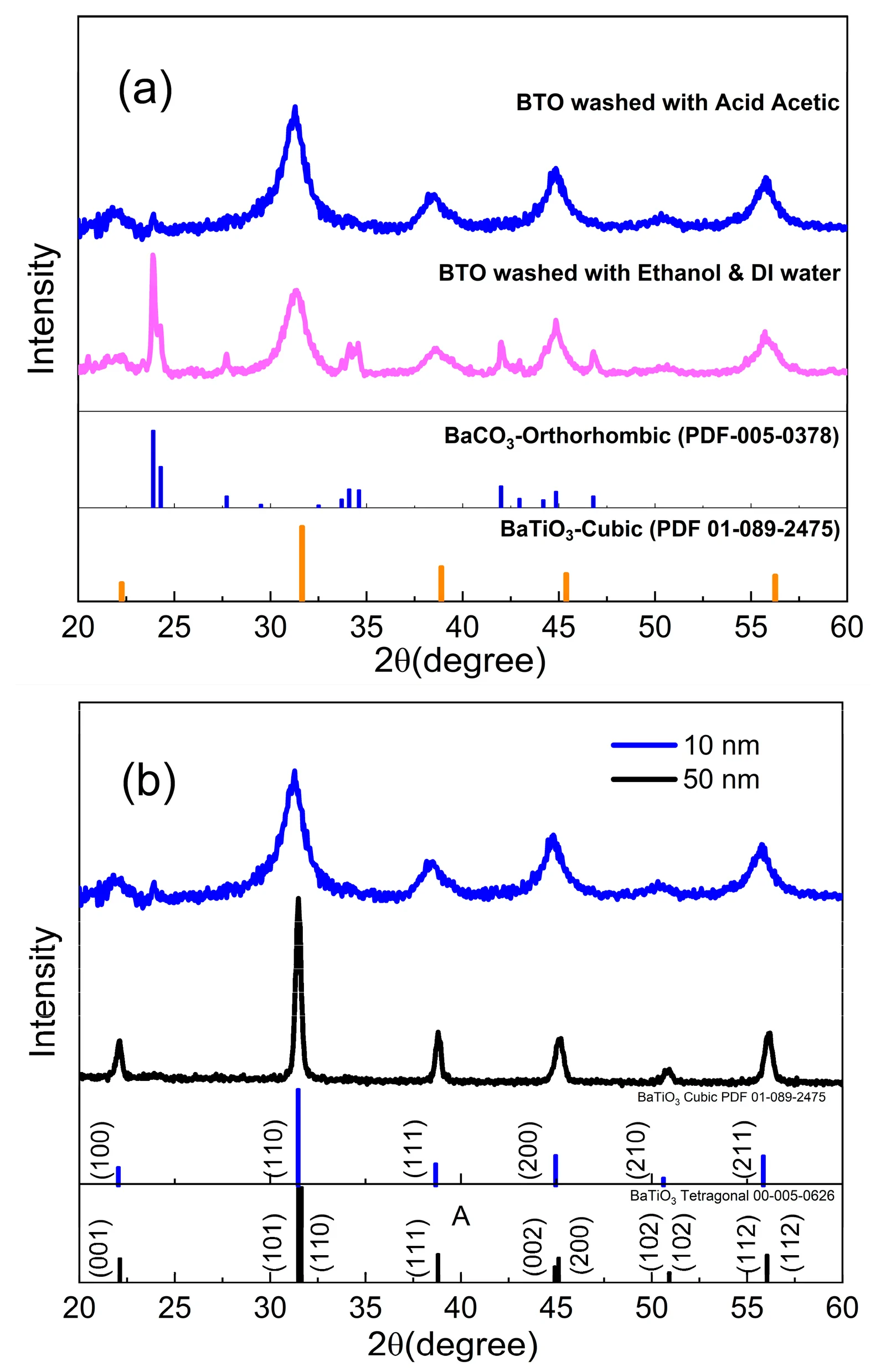

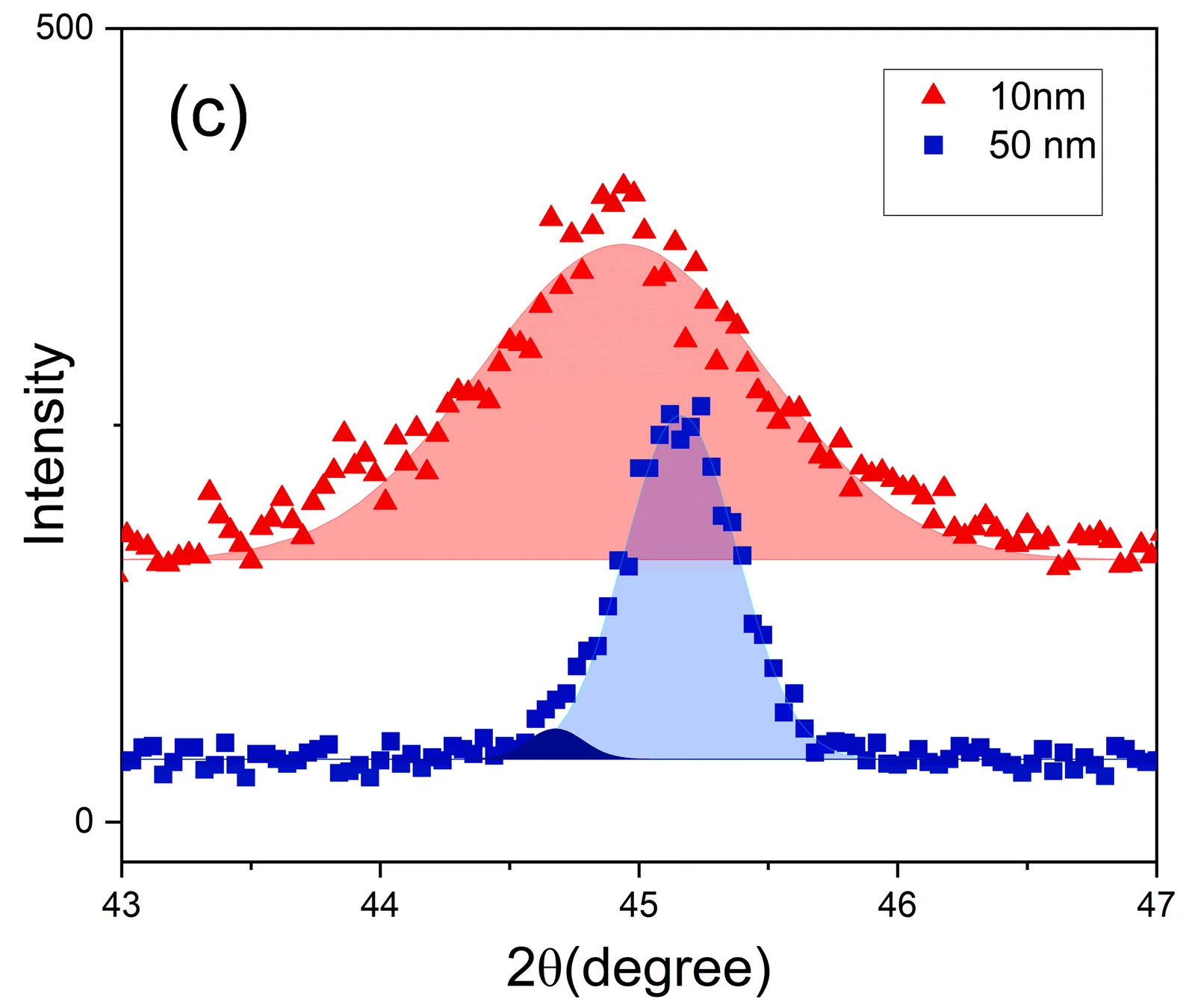
Structural analysis showing (**a**) XRD patterns of the microwave-assisted, hydrothermally synthesized BaTiO_3_-10 nm powders, after washing with ethanol and water and washing with acetic acid, the powder diffraction pattern (PDF) of BaCO_3_ and BaTiO_3_ are also given for comparison (**b**) XRD spectra of BaTiO_3_ nanopowders of different sizes, the PDF of cubic and tetragonal BaTiO_3_ are also given for comparison, and (**c**) high-resolution view of the peak profiles around 45°; the asymmetric peak for the 50 nm powder has been deconvoluted into two Gaussians, while the symmetric peak for the 10 nm powder is fitted with a single Gaussian.

The structural properties of both the commercial BaTiO_3_-50 nm powder and the hydrothermally synthesized BaTiO_3_-10 nm nanoparticles were investigated using XRD. For the 10 nm powder, by comparing the X-ray diffraction spectra with the pattern of the cubic phase of BaTiO_3_, the analysis indicates that the powder is composed mainly of the desired cubic BaTiO_3_, while a secondary phase, identified as barium carbonate (BaCO_3_), is also present. These barium carbonate impurities most likely result from the reaction of barium ions with atmospheric CO_2_ during the synthesis process [25]. The cubic phase of BaTiO_3_ and the BaCO_3_ secondary phase are clearly visible in Figure 1a. These BaCO_3_ impurities are still present even after the samples of BaTiO_3_-10 nm powders underwent three successive washes with ethanol and 5 min of sonication, followed by two washes with distilled water and 5 min of sonication. To remove the secondary phase of BaCO_3_, the 10 nm powder was treated with a 6 mol/dm^3^ acetic acid solution at room temperature for five minutes under ultrasonic agitation [26]. The XRD pattern of the hydrothermally synthesized and subsequently acetic acid-washed sample is shown in Figure 1a, confirming the presence of a purified single-phase of cubic perovskite-type BaTiO_3_. These high-purity BaTiO_3_-10 nm powders were comparable in purity to the commercial samples (exhibiting a very low level of BaCO_3_ impurity). In Figure 1b, the XRD spectra for both 10 and 50 nm powders are presented and correspond to crystallite sizes of 11.8 nm and 53.5 nm, respectively, calculated using the Debye-Scherrer formula and an instrument broadening of 0.06°. The tetragonality of the crystal structure of the BaTiO_3_-50 nm powder can be determined by examining the existence of asymmetry in the peaks, with the shoulder revealing the existence of two peaks, hence two different crystallographic planes. The tetragonal phase of BaTiO_3_ (JCPDF: 05-0626) is given as a reference in Figure 1b. The high-resolution XRD spectra of the peak located at 45 ^o^ shown in Figure 1c can be deconvoluted into two (002) and (200) peaks with a separation of 0.3°, which agrees with the reference of the tetragonal phase of BaTiO_3_. Such asymmetry is not observed for 10 nm sized grains of the nanopowders, in agreement with the reference of the cubic phase of BaTiO_3_. It can therefore be concluded that the 50 nm powder is BaTiO_3_ with a tetragonal crystal structure, while the 10 nm powder is indeed BaTiO_3_ in the cubic phase.

**Figure 2:**
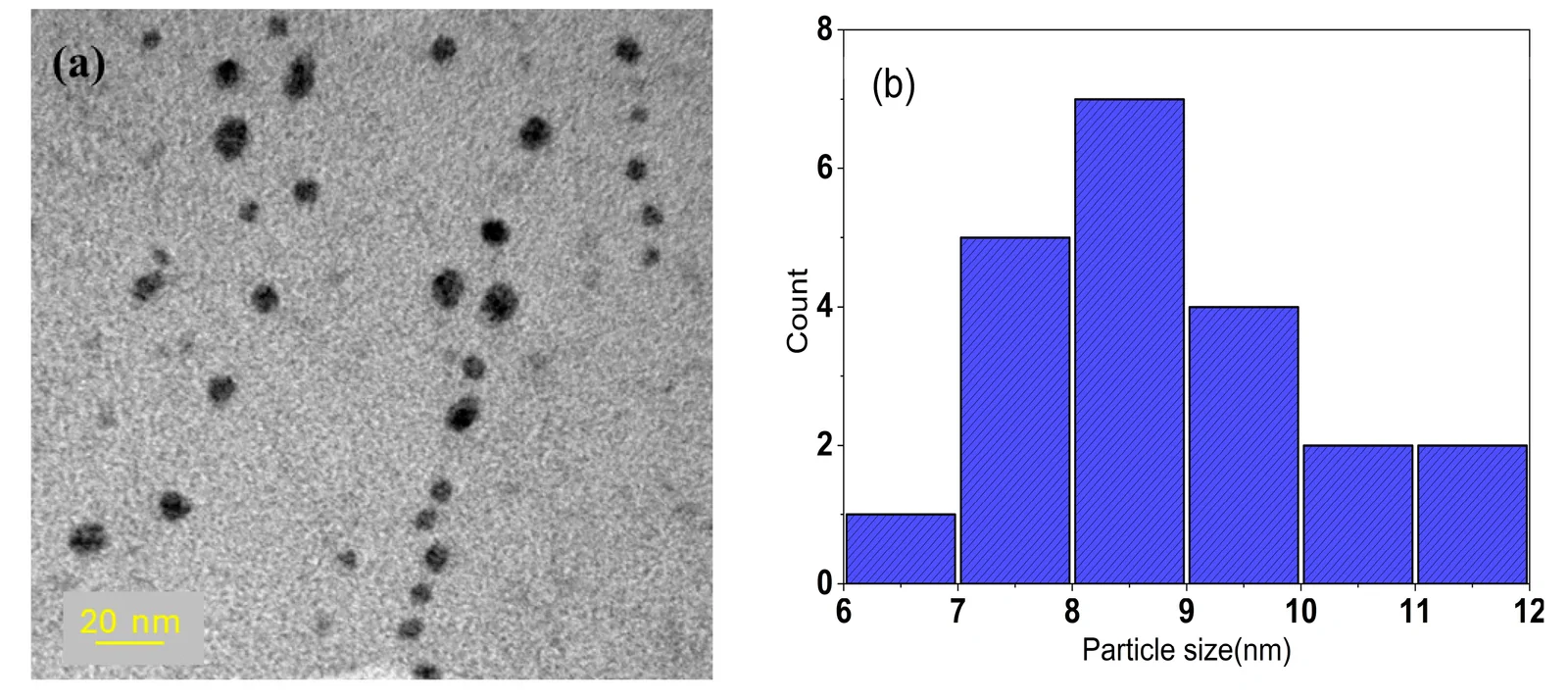

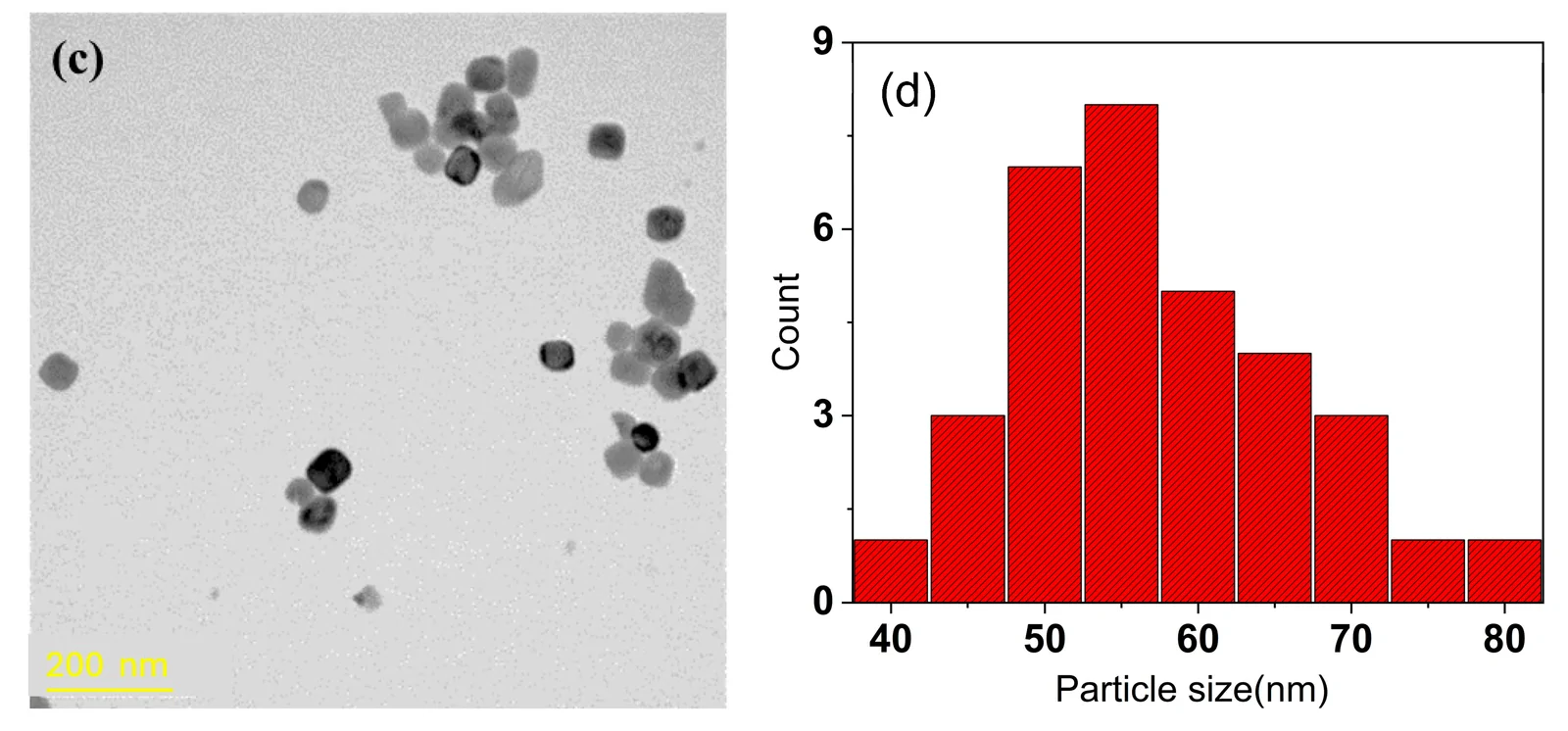
TEM images and particle-size distribution histograms of BaTiO_3_ powders. (**a**) Particles with an average size of 8.5 nm. (**b**) Particle-size distribution histogram corresponding to image (**a**). (**c**) Particles with an average size of 55 nm. (**d**) Particle-size distribution histogram corresponding to image (**c**).

Figure 2a–d shows TEM images of the BaTiO_3_ nanoparticles together with the corresponding particle size distributions obtained from the analysis of these images. In the case of BaTiO_3_-50 nm nanoparticles (Figure 2c), the TEM images show an irregular grain morphology with mostly cubic-shaped habitus. The BaTiO_3_ nanoparticles have a relatively broad size distribution around their nominal size for the two particle sizes investigated. From the histogram shown in the inset of Figure 2c and Figure 2d, the average particle size of these two powders is estimated to be 55 nm and 8.5 nm, respectively.

**Figure 3:**
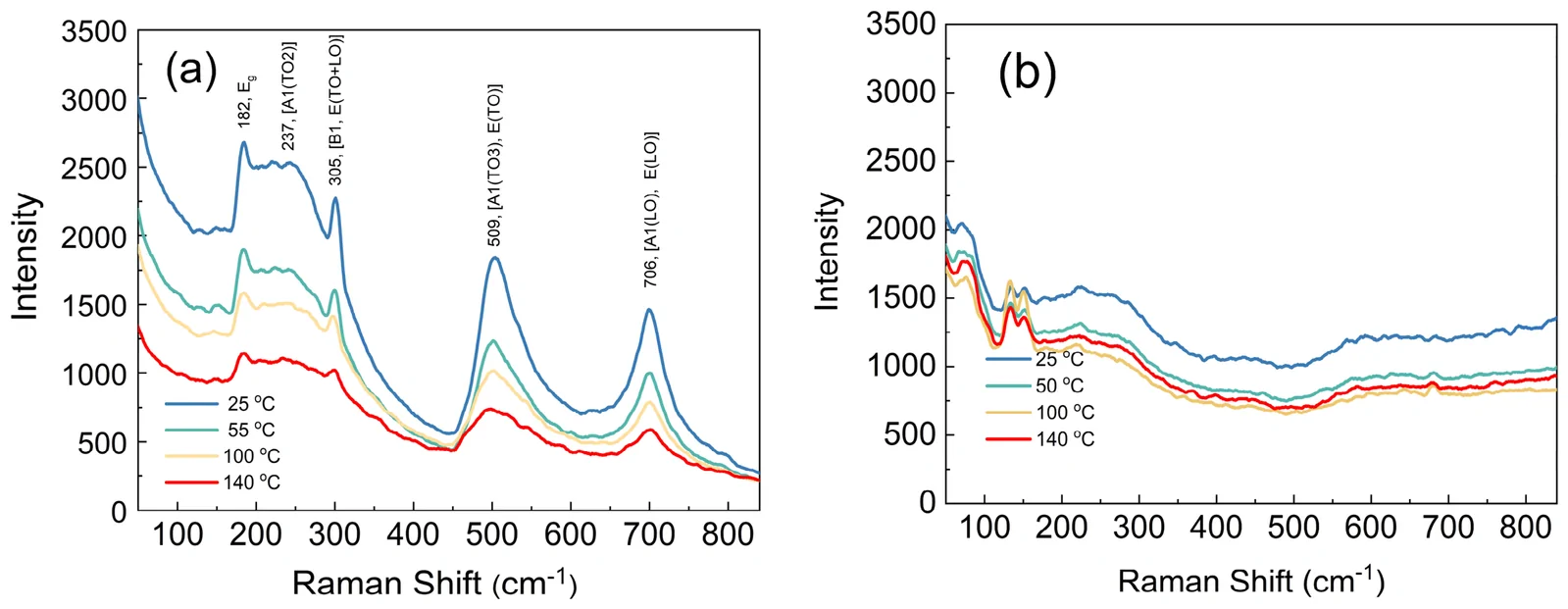
The temperature-dependent Raman spectra of the BaTiO_3_ nanopowder with sizes of (**a**) 50 nm and (**b**) 10 nm. The displayed spectra were smoothed for clarity, without affecting the interpretation of the Raman peaks.

To study the temperature dependence of the crystal structure, and in the absence of variable temperature X-ray diffractometry, the structural properties of the BaTiO_3_ NPs at different temperatures were determined using variable temperature Raman spectroscopy. Figure 3 shows the temperature-dependent Raman spectra for the powders with sizes of 10 and 50 nm. Raman spectra between wavenumbers 50 and 850 cm^−1^ of BaTiO_3_-50 nm nanopowder for temperatures ranging from room temperature to 140 °C are shown in Figure 3a. The Raman spectra of the tetragonal phase of BaTiO_3_ exhibit five different peaks located at 182, 237, 305, 509, and 706 cm^−1^. The two peaks located at 182 and 237 are attributed to the A(TO1) and A(TO2) vibrational modes, respectively. The tetragonal phase of BaTiO_3_ also exhibits characteristic peaks located at 305 and 706 cm^−1^, which are attributed to the B1 + E (TO3 + LO2) and A1 + E (LO3 + LO4) phonon modes related to the Ti–O torsional vibration of the TiO_6_ octahedra. Also, the peak positioned at 509 cm^−1^ is attributed to the A1(TO3) mode. Increasing the temperature causes a strong reduction in these peaks, as can be clearly seen at 140 °C. Figure 3b shows Raman spectra of the 10 nm nanopowder for the same temperature range, all of which are above the bulk tetragonal-cubic phase transition of BaTiO_3_-10 nm. Over the whole temperature range, the Raman spectrum shows no peak in the wavenumber range 200–800 cm^−1^. The cubic phase of BaTiO_3_ being Raman inactive, this is a clear sign that no tetragonal phase is present in the structure of these nanoparticles. The fact that the ferroelectric tetragonal phase is Raman active and that the paraelectric cubic phase of BaTiO_3_ is not makes variable-temperature Raman spectroscopy an effective way to estimate the tetragonal-cubic phase transition temperature. With the help of the 205 cm^−1^ peak extinction, we have estimated the phase transition of BaTiO_3_-50 nm to be around 102 °C. The details of these calculations were given in a previous publication [17]. This value is significantly less than that of the bulk BaTiO_3_, confirming the size dependency of the tetragonal-cubic phase transition temperature. Accordingly, we assume that the phase transition temperature of 10 nm sized nanoparticles is less than 25 °C, which is supported by the fact that no peaks corresponding to a tetragonal phase were observed in either XRD or Raman spectra for these synthetic 10 nm nanopowders [27].

### 3.2. Piezocatalysis Characterization

The objective of this study was to elucidate the roles played by piezoelectric and non-piezoelectric phases in catalytic degradation of organic dyes, employing BaTiO_3_ nanoparticles with varying sizes as nanocatalysts, exhibiting piezoelectric properties or not. The investigation extended the findings of prior research [28], which predominantly assessed the contributions of piezoelectric properties by comparing piezoelectric BaTiO_3_ with the non-piezoelectric TiO_2_ [29,30]. Here, to extract the contributions of the piezoelectric properties to the catalytic reactions, instead of comparing two different materials, we used nanoparticles of the same material as nanocatalysts, namely BaTiO_3_ in its paraelectric and ferroelectric phases. We compared non-piezoelectric cubic BaTiO_3_ with piezoelectric tetragonal BaTiO_3_. The piezocatalytic performance of BaTiO_3_ was evaluated by characterizing the catalytic degradation of MO in an aqueous solution with the BaTiO_3_ catalyst nanoparticles in suspension, and subjecting the solution to various durations of continuous ultrasonic vibrations at a frequency of 40 KHz and a power of 80 W. Following a comprehensive examination of various concentrations, it was determined that the concentration of the catalytic material is pivotal, and that a concentration of 1 g of the catalyst powder per liter yields optimal catalytic performance. Consequently, for all the experimental investigations on the catalytic efficacy of nanocatalyst particle sizes of 10 nm and 50 nm, we used the optimal concentration of catalytic BaTiO_3_ powder in the solution, 1 g per liter.

**Figure 4:**
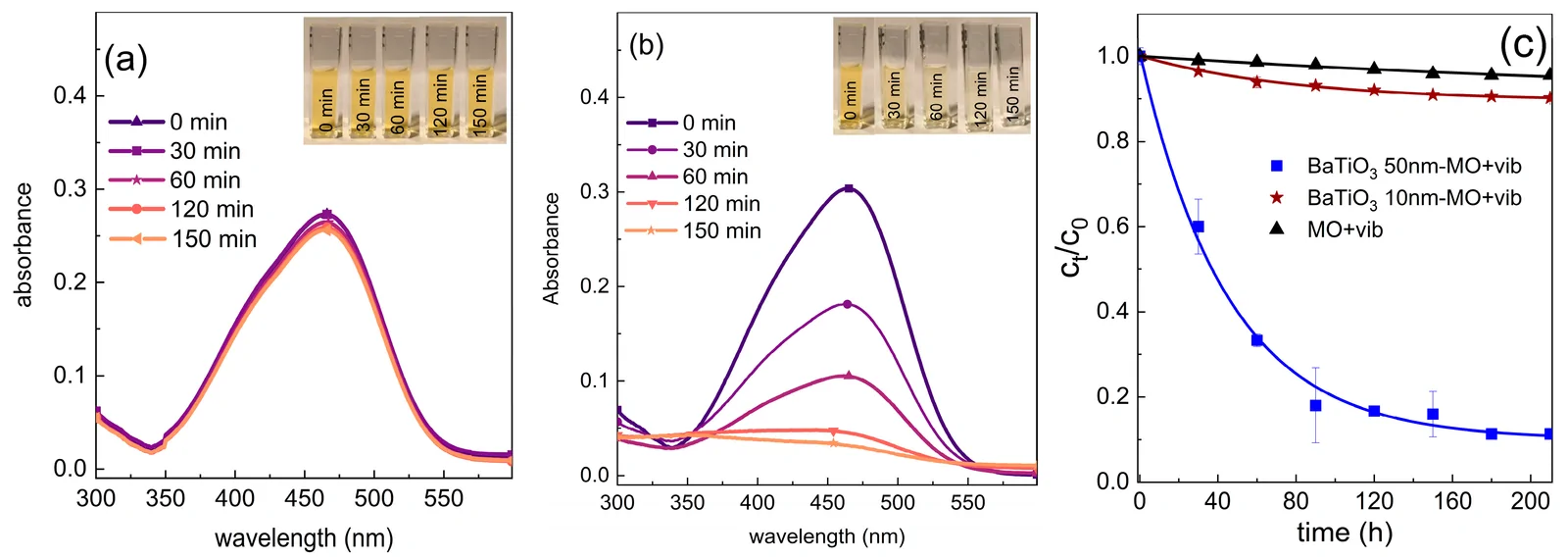
Optical absorption spectra of MO-contaminated water in the presence of the BaTiO_3_ catalyst nanoparticles with sizes of (**a**) 10 nm and (**b**) 50 nm, after different ultrasonication times. The inset of (**b**) shows photographs of the solution after different ultrasonication times. (**c**) Time-dependent catalytic activities of BaTiO_3_ 10 nm and 50 nm nanoparticles for the degradation of methyl orange in solution when submitted to ultrasonic vibrations.

The dependence of the residual dye concentration in the aqueous samples on the ultrasonication duration was assessed, after nanocatalyst removal via sedimentation, through measurements of the optical absorbance of the contaminated solution via UV-Vis spectroscopy. Considering that the magnitude of the absorption peak of the dye is directly related to its concentration in water, the UV-Vis spectra indicate the relative MO concentrations remaining in solution over ultrasonication time for the solutions with both sizes of BaTiO_3_ nanopowder catalysts [31,32]. The absorbance spectra for different ultrasonication times are presented in Figure 4a,b, with their corresponding residual pollutant concentrations (relative to the initial dye concentration) shown in Figure 4c. These datasets were compared against MO-polluted water samples lacking BaTiO_3_ nanopowders. The data clearly show that in comparison to the solution without a catalyst, both piezoelectric and paraelectric BaTiO_3_ nanoparticle suspensions exhibited measurable dye degradation. Remarkably, after 210 min, the piezoelectric BaTiO_3_ nanoparticles with a size of 50 nm degraded approximately 90% of the organic dye molecules. In contrast, the paraelectric BaTiO_3_ nanoparticles with an average size of 10 nm caused only 5% degradation during the same timeframe. Considering that the effective surface of the smaller-sized nanoparticles is approximately 5 times larger than that of the 50 nm sized BaTiO_3_ nanopowder, the difference in catalytic activity per unit surface is even more significant. This huge difference in catalytic activity between the 10 nm cubic BaTiO_3_ nanoparticles and the 50 nm tetragonal BaTiO_3_ nanoparticles, despite the relative differences in surface area, strongly suggests the involvement of piezoelectric catalysis [33,34,35]. To determine the reaction rate of BaTiO_3_ nanopowders’ catalytic reactions, the relative concentration (C_t_/C_0_) function of each curve was fitted using the Langmuir−Hinshelwood model of the kinetic reaction rate, under the assumption of low MO concentrations [36]. The model then simplifies into a pseudo-first-order kinetic model expressed as −ln (C_t_/C_0_) = K_eff_. The experimental result of −Ln (C_t_/C_0_) vs. ultrasonication time follows quite well the linear relationship of the model, the slope of which determines the reaction rate.

**Figure 5:**
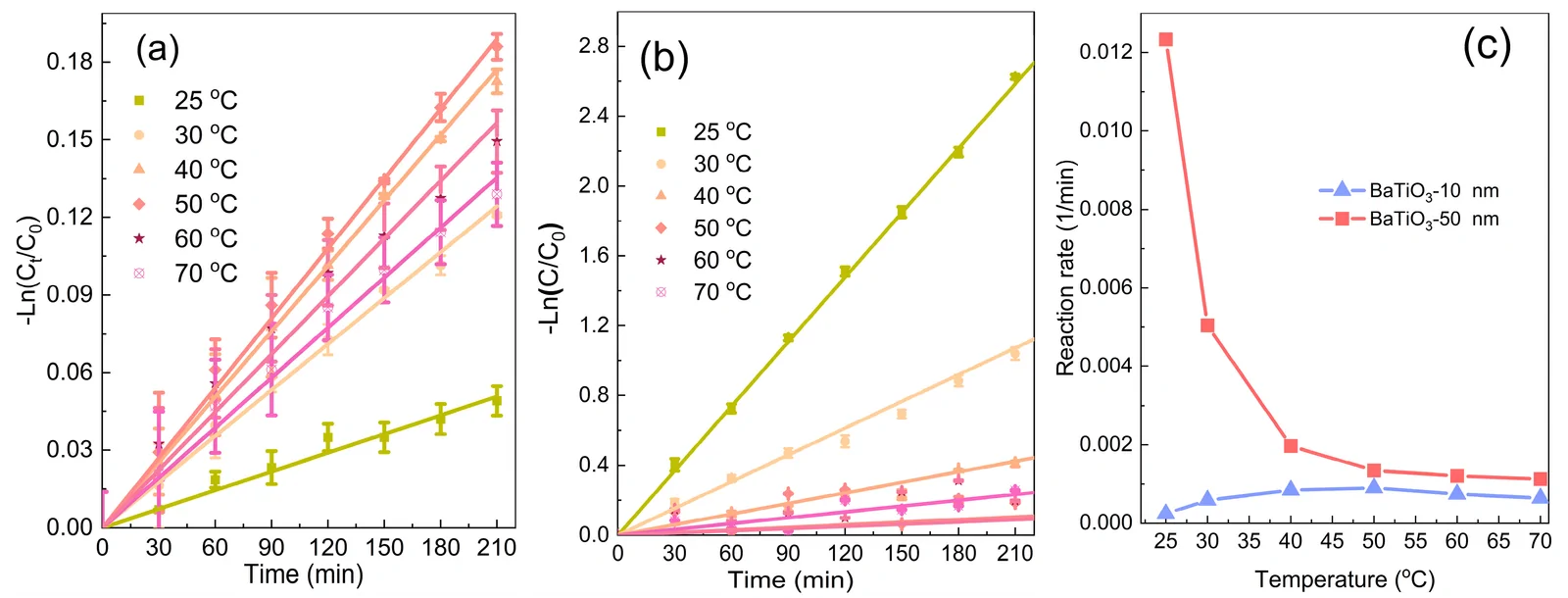
Exponential decrease of the MO relative concentration: (−Ln (C_t_/C_0_) vs. ultrasonication time linear dependence) for the BaTiO_3_ nanopowders with the size of (**a**) 10 nm, and (**b**) 50 nm, at different temperatures. (**c**) Temperature dependence of the reaction rate calculated from (**a**,**b**). (Error bars in (**b**) are present but too small to be clearly seen).

Figure 5a,b depict the MO degradation reaction rates for several temperatures for (a) BaTiO_3_-10 nm nanopowders, and (b) BaTiO_3_-50 nm nanopowders. It is observed that the catalytic activity of the BaTiO_3_-10 nm nanopowders is only weakly dependent on temperature (Figure 5a). For the 10 nm nanocatalysts, increasing the reaction temperature from room temperature to 50 °C results in a slight enhancement of the catalytic activity, whereas a further increase in temperature leads to a gradual decline in the reaction rate. This trend is consistent with the typical temperature dependence reported for sonocatalytic systems [37]. At lower temperatures, the reduction in water viscosity and the increase in molecular diffusion and reaction kinetics facilitate the catalytic process. However, at higher temperatures, the increase in the vapor pressure of water weakens acoustic cavitation by cushioning bubble collapse, thereby reducing the intensity of shock waves, microjets, and the generation of reactive oxygen species. Consequently, the beneficial effect of enhanced reaction kinetics is progressively outweighed by the reduction in cavitation intensity, leading to a decrease in the overall catalytic activity. In sharp contrast with the weak temperature dependence of the BaTiO_3_-10 nm nanopowders, the reaction rate of the BaTiO_3_-50 nm nanopowders decreases significantly with a relatively small increase in temperature (Figure 5b). Figure 5c illustrates the temperature dependence of the reaction rates obtained from Figure 5a,b vs. temperature for both BaTiO_3_-50 nm and BaTiO_3_-10 nm nanoparticles. According to these findings, the 50 nm powders of piezoelectric BaTiO_3_ exhibit a much higher (approx. 10 times higher) catalytic reaction rate at room temperature than the paraelectric BaTiO_3_-10 nm nanopowder, as well as a very strong temperature dependence, with the reaction rate quickly decreasing with temperature [38,39]. This strong decrease in catalytic response is consistent with the decrease in the spontaneous polarization, and it turns off the piezoelectric properties of the BaTiO_3_-50 nm nanoparticles with increasing temperature. It has been previously reported that the specific temperature dependence of piezocatalytic response corresponds closely to the temperature dependence of the d_15_ piezoelectric coefficient of BaTiO_3_ [40]. Above the Curie temperature, the catalytic activity of the BaTiO_3_-50 nm nanoparticles reaches the same level as the non-piezoelectric BaTiO_3_-10 nm nanoparticles. The low reaction rate observed for the BaTiO_3_-10nm nanoparticles at all temperatures can be attributed to their absence of spontaneous polarization in the paraelectric phase (hence the absence of piezoelectricity), as evidenced by the Raman spectroscopy results shown in Figure 3a,b. The absence of piezoelectric properties in the 10 nm BaTiO_3_ particles reduces the generation and separation of reactive charge carriers on the nanoparticle surface, leading to a lower production of reactive oxidative species. Consequently, the catalytic activity is low despite the higher specific surface area and greater number of potentially active surface sites associated with the smaller particle size.

**Figure 6:**
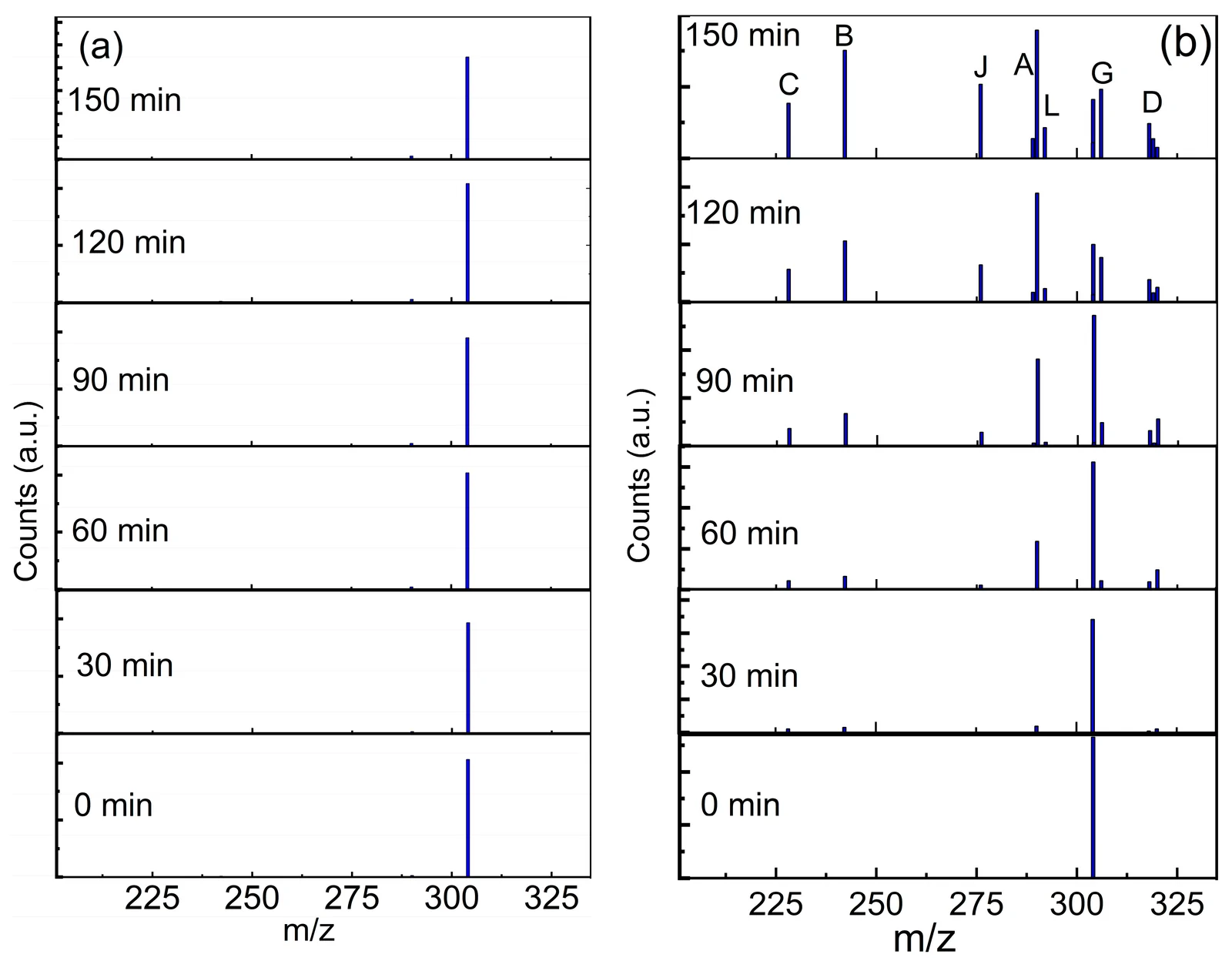
LC-MS results obtained for the MO-contaminated water when ultrasonicated for various times in the presence of piezoelectric BaTiO_3_ nanoparticles as a catalyst with sizes of (**a**) 10 nm and (**b**) 50 nm. Letters in (**b**) correspond to chemical structures given in Figure 7.

Furthermore, liquid chromatography–mass spectrometry (LC–MS) was employed to study the reaction pathways involved in MO degradation under piezo- or sonocatalytic conditions. Figure 6a and Figure 6b show the MS spectra of samples treated with 10 nm and 50 nm BaTiO_3_ nanoparticles, respectively.

For the BaTiO_3_-10 nm catalyst (Figure 6a), the mass spectra exhibited no major changes, except for a slight decrease in the main MO peak at *m*/*z* 304. Some byproducts appeared within 150 min. characterized by low-intensity peaks, which then disappeared during or after ultrasonication, possibly leading to CO_2_ and H_2_O. This indicates a very limited degradation of MO. In contrast, the MO-contaminated solution with BaTiO_3_-50 nm nanocatalyst (Figure 6b) showed pronounced degradation, with a 99.4% reduction in the intensity of the MO peak after 150 min. While the main peak at *m*/*z* 304 decreased, new peaks at *m*/*z* 290, 276, 242, and 228 emerged, corresponding to smaller intermediate degradation products, with the most intense peaks being clearly observed while others remain weaker. Each of these peaks has been marked with a specific letter shown in Figure 6b to follow their time dependence.

**Figure 7:**
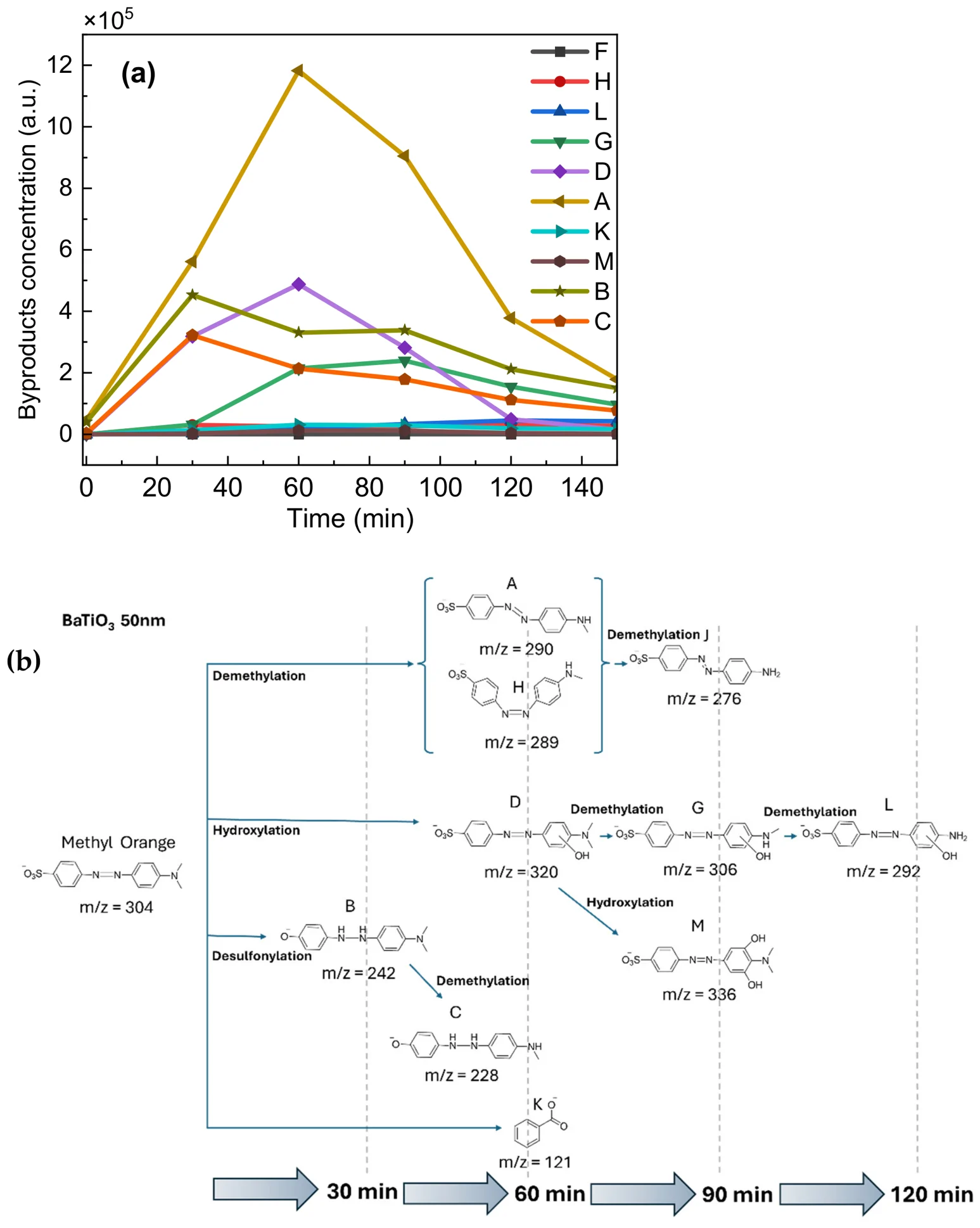

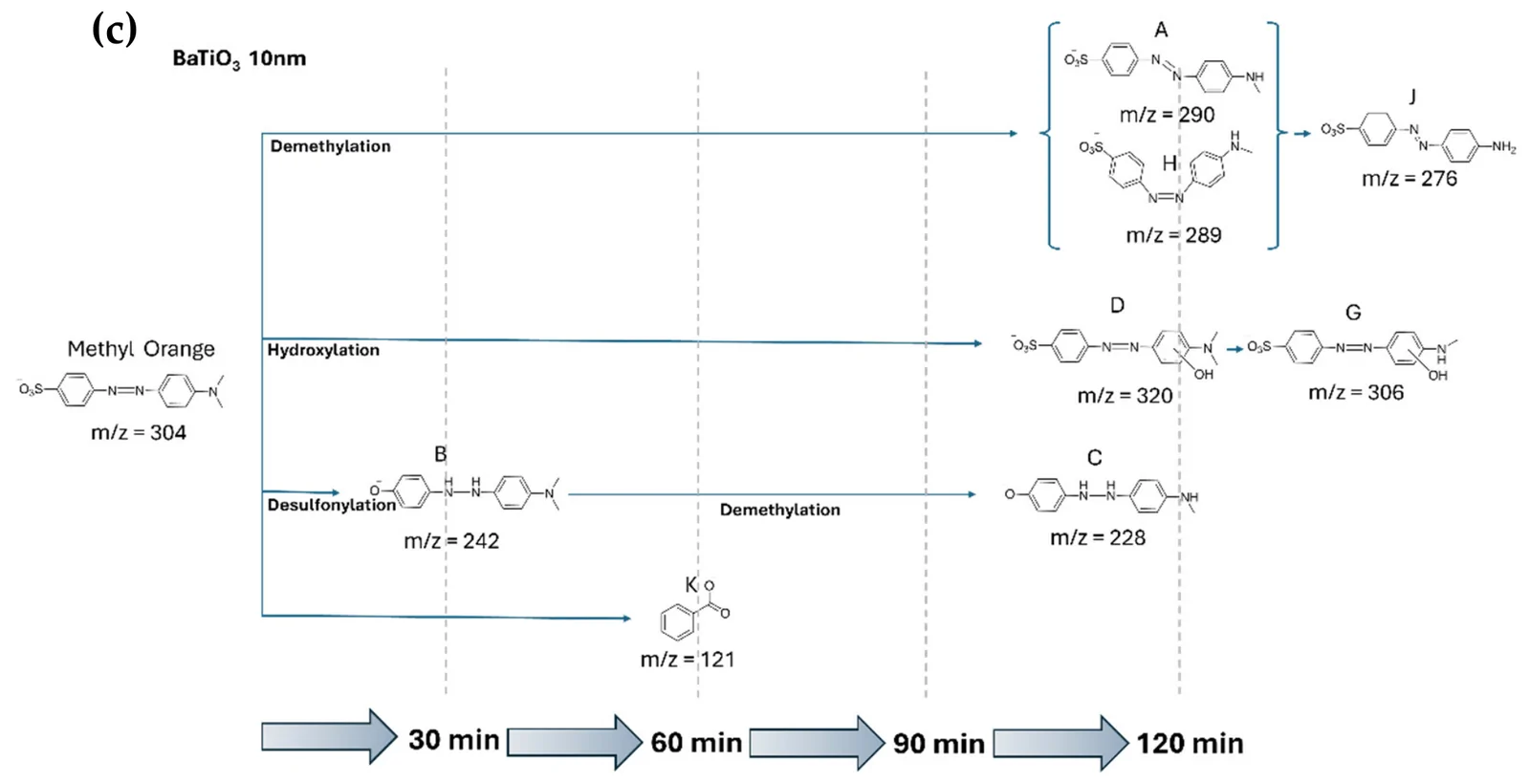
(**a**) A view of the concentration of degradation byproducts when BaTiO_3_-50 nm is used as a catalyst. Schematic illustration of the proposed degradation byproducts (labeled with letters) pathway of MO degradation when ultrasonicated and when (**b**) BaTiO_3_-50 nm and (**c**) BaTiO_3_-10 nm are used as a catalyst.

Reactive radicals such as •OH and •$O_{2}^{-}$ are responsible for the degradation processes [41,42]. Figure 7 shows possible pathways for the redox reactions involved in the initial stages of the MO degradation, leading to the byproduct molecules observed. The 50 nm nanoparticles demonstrate a rapid degradation (Figure 7b). Products A, B, C, and D are the first byproducts formed, with A showing the highest intensity, B and C also showing strong intensities, and D showing moderate intensity. These products are formed through one of three pathways: desulfonylation (product B), demethylation (products A and H), and hydroxylation (product D) [43]. All three pathways are consistent with the formation of hydroxyl and superoxide radicals during the catalytic reaction. The mass spectra demonstrate that as ultrasonication time increases, the degradation of MO molecules and A, B, C, and D continues, with these intermediates first increasing in concentration (up to around 60 min) and then decreasing, producing smaller molecules such as J at earlier stages and G at later stages. Molecule L was only detectable by LC-MS after 90 min of ultrasonication, indicating it is likely a later-stage (secondary or tertiary) degradation product, formed after the decay of earlier intermediates [40]. This sequential formation and decomposition of intermediate byproducts should continue until only small molecules, most likely H_2_ and CO_2_, remain as final products. For the MO-contaminated solution with BaTiO_3_-10 nm nanoparticles, only minimal amounts of degradation products can be detected, and those are only apparent at long exposure times (Figure 7c). However, the products that are eventually formed are identical to those observed for BaTiO_3_-50 nm, suggesting that the active radicals might be similar for both nanoparticle sizes, but that the piezocatalytic activation is much more efficient for BaTiO_3_-50 nm [44].

This catalytic degradation of MO was observed exclusively for nanoparticles where ferroelectric BaTiO_3_ was used as a catalyst, whereas nanoparticles of paraelectric BaTiO_3_ or non-piezoelectric materials, such as TiO_2_, exhibited much less catalytic activity [17]. These findings confirm the superior catalytic efficiency of larger BaTiO_3_ particles, attributed to their good piezoelectric properties, which promote free radical generation under ultrasonication [45].

## 4. Conclusions

In this study, by taking advantage of the size-dependence of the spontaneous polarization of BaTiO_3_ versus temperature, we compared the catalytic degradation reactions of the dye MO under ultrasonication for piezoelectric BaTiO_3_-50 nm, which exhibit a tetragonal crystal structure, and for non-piezoelectric BaTiO_3_-10 nm nanoparticles with a cubic crystal structure. It was observed that piezoelectric 50 nm sized nanoparticles had much better catalytic reaction rates under ultrasonication at room temperature than non-piezoelectric 10 nm nanoparticles. Centrosymmetric cubic BaTiO_3_ lacks spontaneous polarization, therefore exhibiting no piezoelectricity. The non-piezoelectric BaTiO_3_ does, however, exhibit some catalytic activity under ultrasonication that is attributed to sonocatalysis. In the case of catalyst materials with piezoelectric properties (i.e., 50 nm powder), it was observed that (i) the MO degradation reaction rates are much higher than for the non-piezoelectric nanocatalysts, strongly enhanced by the piezoelectric properties of BaTiO_3_, and (ii) that the piezocatalytic response strongly decreased with temperature, down to, the Curie temperature at which the tetragonal to cubic structural transition takes place and the ferroelectric and piezoelectric properties of BaTiO_3_ vanish. This strong decrease in catalytic response is consistent with the decrease in spontaneous polarization and piezoelectric properties, and its specific temperature dependence corresponds to the temperature dependence of the d_15_ piezoelectric coefficient of BaTiO_3_ [40]. Above the Curie temperature, the catalytic activity reaches the same level as the non-piezoelectric BaTiO_3_-10 nm nanoparticles. Considering the room temperature catalytic reaction rates recorded for both the BaTiO_3_-50 nm and the BaTiO_3_-10 nm, the contribution of the piezoelectric properties to the catalytic activity, defining the *piezocatalytic activity*, is estimated to be about 90% of the overall catalytic activity, the remaining 10% being due to sonocatalysis (at the low nanocatalyst concentrations used in this study, the contribution of tribocataylsis has been estimated to be negligible). LC–MS results further confirmed that the MO degradation proceeded through successive radical-mediated steps, with a higher degree of intermediate transformation observed for the BaTiO_3_-50 nm nanoparticles. The sequential formation and decomposition of intermediates continued until only small molecules remained as the final products (with *m*/*z* less than 118 for the ultrasonic durations used in the presented experiments). The results further confirmed that for the BaTiO_3_-50 nm nanoparticles, the concentration of MO decreased by 99.4% after 150 min, indicating nearly complete degradation. We have therefore demonstrated that using a catalyst with good piezoelectric properties at the operating temperature greatly increases the catalytic activity in sonochemistry reactions.

## Data Availability

The original contributions presented in this study are included in the article. Further inquiries can be directed to the corresponding author.
